# Impact of progressive familial intrahepatic cholestasis on caregivers: caregiver-reported outcomes from the multinational PICTURE study

**DOI:** 10.1186/s13023-022-02177-0

**Published:** 2022-02-02

**Authors:** Claudia Mighiu, Sonia O’Hara, Enrico Ferri Grazzi, Karen F. Murray, Jörn M. Schattenberg, Emily Ventura, Melanie Karakaidos, Alison Taylor, Harpreet Brrang, Anil Dhawan, Jose Willemse, Alan Finnegan

**Affiliations:** 1 HCD Economics, Daresbury, WA4 4FS UK; 2 Pediatric Institute, Cleveland Clinic Children’s Hospital, Cleveland, OH USA; 3 University Medical Center of the Johannes Gutenberg-University, Mainz, Germany; 4 PFIC Advocacy and Resource Network, Stanton, KY USA; 5 Children’s Liver Disease Foundation, Birmingham, UK; 6 Pediatric Liver GI and Nutrition Center, King’s College Hospital, London, UK; 7 Dutch Liver Patient Association (NLV), Hoogland, The Netherlands; 8 Faculty of Health and Social Care, University of Chester, Chester, UK

**Keywords:** Progressive familial intrahepatic cholestasis, Caregiver burden, Work productivity, Employment, Patient-reported outcomes

## Abstract

**Background:**

Progressive familial intrahepatic cholestasis (PFIC) is a spectrum of rare genetic diseases characterized by inadequate bile secretion that requires substantial ongoing care, though little research is published in this area. We report health-related quality of life (HRQoL) and work productivity outcomes from the retrospective, cross-sectional PICTURE study investigating the burden of PFIC on caregivers. Information from caregivers of patients with PFIC 1 or 2 in Germany, the United Kingdom and the United States from September 2020 to March 2021 was included.

**Results:**

The PICTURE study sample comprised HRQoL responses from 22 PFIC caregivers. Patients were on average 8.2 years old; most caregivers were 30–49 years old (68%) and mothers (77%). Median CarerQoL-7D score was 67.7/100; mean CarerQoL-VAS score for general happiness was 5.7/10 (SD 2.1). Most caregivers reported fulfilment in their caregiving responsibilities, but problems with mental and physical health, finances, and relationships. When stratified by patient’s PFIC type, mean CarerQoL-7D and CarerQoL-VAS scores suggested worse HRQoL outcomes with PFIC2 versus PFIC1 (59.4 vs. 71.2, and 5.3 vs. 6.5, respectively). Additionally, more caregivers reported impact on sleep in the PFIC2 versus PFIC1 subgroup (93% vs. 75%). When stratified by history of PFIC-related surgeries, mean CarerQoL-7D and VAS scores were higher among those whose children had no specified surgeries (67.7 vs. 59.0/100 and 6.2 vs. 5.2/10, respectively). Nearly all caregivers reported an impact of caregiving responsibilities on sleeping (86%) and on personal relationships (82%). No caregivers reported having formal care support. Most caregivers were employed (73%); a third reported mean productivity loss of 12.9 days (SD 19.3) over the last 3 months, and a mean of 2.8 (SD 9.5) missed years of employment during their career. A higher number of workdays were missed by PFIC 2 caregivers compared to PFIC1 over last 3 months (16 days vs. 3 days).

**Conclusions:**

The PICTURE study has demonstrated the prevalent, comprehensive, and meaningful burden that caring for an individual with PFIC has on caregivers. Despite fulfilment from caregiving, the breadth and depth of these responsibilities reduced caregiver reported HRQoL including mental and physical health, productivity, career prospects, sleep, relationships and finances.

**Supplementary Information:**

The online version contains supplementary material available at 10.1186/s13023-022-02177-0.

## Background

Progressive familial intrahepatic cholestasis (PFIC) is a spectrum of rare genetic diseases of cholestasis, characterized by inadequate bile secretion, often requiring liver transplantation and leading to liver failure and early death [1–7]. The rare nature of PFIC has made reliable epidemiological data difficult to establish, with the estimated prevalence most often reported to fall between 1/50,000 and 1/100,000 people [6, 8]. The prevalence of PFIC in children with liver conditions ranges from 9–13% of those with cholestasis, splenomegaly, or acute liver failure [8–11]. Symptoms tend to present in infancy (≤ 3 months old), most commonly including jaundice and pruritus, with severe pruritus reported in 76–80% of patients [3, 4, 7]. Mortality estimates for patients with PFIC vary based on liver transplantation outcomes, presence of hepatocellular carcinoma, and other common complications such as liver failure, infections, and internal bleeding, but it is estimated to be 87% for untreated PFIC [6, 8].

The most prevalent PFIC types within the patient population are PFIC type 1 and type 2. Type 1 (PFIC1) presents in infancy or early childhood and is caused by mutations in the ATP8B1 (or FIC1) gene. PFIC1 typically manifests with a moderate to severe intensity: children may have severe itching (pruritus), an enlarged liver (hepatomegaly) and may also have extrahepatic symptoms, including watery diarrhea, pancreatitis, deafness and short stature or failure to thrive [3]. Children with PFIC1 may experience end-stage liver disease (ESLD) within the first decade of life.

Type 2 (PFIC2) is caused by a malfunctioning copy of the gene ABCB11 (encoding the BSEP export pump). Also typically diagnosed in early childhood, PFIC2 shows rapid disease progression, with severe itching, hepatomegaly, and a risk for hepatocellular carcinoma and cholesterol stones; extrahepatic manifestations are more rarely encountered in PFIC2 patients [3].

The rare nature of PFIC has presented challenges to understanding its impact on the daily lives of patients and their caregivers. Recent systematic reviews have identified few studies investigating the health-related quality of life (HRQoL) of patients with PFIC [8, 12]. HRQoL scores using the PedsQL 4.0 Generic Core Scale have been reported to be lower for children with PFIC compared with healthy peers (73 vs. 84/100 points) and for their respective parents (79 vs. 82/100) [13]. Lind and colleagues [14] reported worse overall HRQoL among children with PFIC who underwent partial external biliary diversion compared with healthy peers (PedsQL, 57 vs. 83/100) Findings were similarly worse for physical and psychosocial domains, as well as for parent proxy assessments of the child’s HRQoL. Strong, significant correlation of HRQoL with severity of pruritus was also reported [14].

In the context of extremely limited information on the humanistic burden of PFIC on patients directly, there is no identifiable published work on the burden of PFIC on their caregivers. Considering the early onset, severe nature, and certain morbidity associated with PFIC through the lifespan, the care requirements of these patients are necessarily substantial and persistent. Analogous work has shown significant caregiver burden including reduced HRQoL and work productivity among parents of children and adolescents with cystic fibrosis (another rare, genetic, early-onset condition with common organ transplantation and increased risk of mortality) [15–17]. To this end, the retrospective, cross-sectional PICTURE study was designed to investigate the clinical, humanistic and economic burden experienced by patients with PFIC and their caregivers, as well as societal costs of PFIC [18]. This paper addresses the self-reported HRQoL and work productivity outcomes captured by the PICTURE study, providing a better understanding of the impact of PFIC on caregivers in particular.

## Methods

A retrospective analysis of data from the PICTURE study was conducted, the design and methods of which have been reported previously [18]. The PICTURE study was a cross-sectional burden of illness study of physician and caregiver-reported information for patients with PFIC type 1 or 2 in France, Germany, the United Kingdom and the United States from September 2020 through to March 2021. While the PICTURE study captured information related to the clinical, humanistic and economic burden of PFIC, this paper’s analysis focuses on the caregiver-reported humanistic burden of PFIC.

The PICTURE study design has two main sources of data: physicians and caregivers. Physicians provided clinical and resource use data of PFIC patients at the time of consultation, via an electronic Case Report Form (eCRF). Caregivers of PFIC patients, recruited by the physician as they attended a clinical appointment with the patient, completed online specific self-completion questionnaires about the impact of the disease on their lives.

The study was conducted under the guidance and expertise of an Expert Reference Group (ERG), consisting of a representative of academia as principal investigator (University of Chester), partnering charity and advocacy representatives (PFIC Network and Children’s Liver Disease Foundation), as well as experts in the field of liver diseases: Prof. Karen Murray, Prof. Jorn Schattenberg, Prof. Anil Dhawan and Jose Willemse.

The study protocol and materials were approved by the Research Ethics Sub-Committee of the Faculty of Health and Social Care at the University of Chester (no. RESC0820-1042). The study was conducted in accordance with all relevant ethical guidelines; all caregivers provided informed consent.

### Participants

Participant outreach and recruitment was conducted via electronic media, using both a standard fieldwork approach and a hybrid approach. The standard fieldwork approach was conducted from September 2020 through to March 2021 and aimed to recruit representative samples of physicians treating PFIC in each country (including hepatologists, gastroenterologists, general practitioners, pediatricians, and medical geneticists). Eligible physicians had to have ≥ 2 years of practice experience and have treated ≥ 1 patient with PFIC in the last 12 months. Participating physicians recruited consecutive eligible patients/caregivers (maximum of 10) during routine clinical visits for any reason, including adult caregivers/guardians of patients (any age) with genetic diagnosis of PFIC (subtypes 1, 2 or 3) ≥ 12 months prior to study entry. Patients/caregivers were excluded for current or recent (≤ 12 months) clinical trial participation. Physicians completed electronic case report forms (eCRFs) with clinical and economic information based on the patient’s medical history. Caregivers completed online questionnaires regarding the impact of PFIC on their lives and productivity. Physicians performed all activities outside of professional practice parameters and received remuneration based on fair market value; caregivers also received remuneration for completion of questionnaires.

The hybrid recruitment approach was conducted through February and March 2021 to bolster caregiver participation, as their involvement was likely affected by the COVID-19 pandemic over 2020 fieldwork months. Partnerships with the PFIC Network (pfic.org) and the Children’s Liver Disease Foundation (CLDF; childliverdisease.org) facilitated direct outreach to families of patients with PFIC. Participants recruited through the hybrid approach completed the same online questionnaire on the impact of PFIC as in the fieldwork approach, with 5 additional questions related to clinical outcomes and resource use, to facilitate analyses of clinical and economic burden in the absence of eCRFs completed by the physicians.

Given that PFIC is a rare disease, measures were taken to solidify the investigators’ confidence that the study sample was based on patients identified with PFIC. An interim quality-control analysis of incoming data revealed that a high percentage of cases reported were adults, with no surgical history (no partial biliary diversion [PBD] or liver transplantation). This suggested that non-progressive cholestasis cases were included for participation in the study. Therefore, a clinical algorithm was developed and applied post-hoc, to select children and young adults most likely to exhibit PFIC. The requirements were patients < 5 years old at the time point of PFIC diagnosis and < 18 years old at the time of study entry. Exceptions were made for those aged 18–35 at study entry and ≤ 5 years old at diagnosis if they had a record of a PFIC-related surgery such as PBD or liver transplantation (Additional file 1: Fig. S1). Otherwise, older patients (diagnosed > 5 years of age and/or aged > 35 years at study entry) were not likely to have had PFIC or to have survived PFIC without a record of life-sparing surgery.

### Variables and outcomes

Patient and caregiver characteristics were provided by the physicians and caregivers (fieldwork approach) or caregivers only (hybrid approach), including age, sex, country, PFIC subtype, caregiver type (i.e., mother, father), highest caregiver education level, marital status, and urbanicity (rural, village, town, city). Caregivers provided information about the impact of PFIC on HRQoL, work productivity, their professional career, sleep, and relationships. A supplemental question regarding the impact of the COVID-19 pandemic on PFIC caregiving was also included.

Caregiver-reported HRQoL was captured using the validated 7-dimension CarerQoL-7D and the caregiver HRQoL visual analog scale (CarerQoL-VAS; Institute for Medical Technology Assessment, The Netherlands; www.imta.nl/carerqol/) [19, 20]. The 7 dimensions of the CarerQoL-7D indicate 3-level responses (“no”, “some”, or “a lot of”) for 2 ‘positive’ dimensions regarding level of fulfilment carrying out care tasks and availability of support when needed to carry out care tasks, and 5 ‘negative’ dimensions related to relationship problems with the care receiver, problems with the caregiver’s own mental health, financial problems due to care tasks, and problems with the caregiver’s own physical health [20]. A weighted average utility score based on UK utility weights [21] (Additional file 1: Table S1) was calculated on a 0–100-point scale where 0 represents the “worst possible caregiving situation” and 100 represents the “perfect caregiving situation” (a lower weighted score indicated greater caregiver burden). For the CarerQoL-VAS, responders marked a continuous 0–10 scale indicating how they felt at the moment, from “completely unhappy” (0) to “completely happy” (10).

Work productivity was assessed using the Work Productivity and Activity Impairment questionnaire Adapted for Companion or Caregiving v2.0 (WPAI; www.eprovide.mapi-trust.org). The WPAI captures the percentage of impairment in the past 7 days, in terms of work-related absenteeism (not being present at work), presenteeism (present at work, but with productivity limitations), work productivity loss (based on absenteeism and presenteeism), and impairment of regular activities due to health-related caregiver duties, where higher scores indicate greater impairment, or worse outcomes [22]. Further productivity impact was assessed via employment status, impact on work in the past 3 months (missed workdays or productivity impact), stopping work due to caregiving (missed work years), and impact on career building (alternate career choice, prevention of career progression, prevention from working more hours).

Caregivers reported the impact of caregiving on their sleep (no, some, moderate, or severe difficulty sleeping, or hardly ever sleep) and relationships (no effect, affected relationship with partner, affected relationship with other family members, affected relationship with friends). Results were examined overall, by PFIC type (1 or 2), by history of surgery (with PBD/Liver transplant vs. without PBD/Liver transplant), and by history of specific procedures (PBD, Liver transplant, both, neither).

Caregivers also indicated their ability or lack thereof to access supplementary professional care. The impact of the COVID-19 pandemic was also assessed, in relation to the impact on resource use, caregiver mental health, and caregiver physical health.

### Statistical analysis

All data were summarized using descriptive statistics including frequencies, proportions, and measures of central tendency using STATA 16 (www.stata.com). No imputation of missing values was performed.

## Results

The initial PICTURE study sample comprised 320 eCRFs from participating physicians, of which 214 (67%) were excluded by the clinical algorithm exercise to identify the most probable PFIC cases (n = 106). HRQoL responses were available from a total of 22 caregivers (fieldwork approach, n = 7, 32%; hybrid approach, n = 15, 68%). Caregivers were evenly distributed across Germany, the UK and US. Most caregivers were 30–49 years old (68%) and mothers (77%; Table 1). Approximately half of the patients were 0–9 years old (59%; mean 8.2 years), and female (59%; Additional file 1: Table S2).

**Table 1:** Caregiver demographic characteristics

Caregiver characteristics (n = 22)	N (%)
Age	
20–29	4 (18)
30–39	9 (41)
40–49	6 (27)
50–59	3 (14)
Caregiver type	
Mother	17 (77)
Father	5 (23)
Country	
Germany	8 (36)
United Kingdom	7 (32)
United States	7 (32)
Education level	
Secondary school	2 (9)
High school	4 (18)
University, bachelor	10 (46)
University, masters	4 (18)
None	1 (5)
Other	1 (5)
Marital status	
Married	19 (86)
Single	2 (9)
Separated/divorced	1 (5)
Area of residence	
Rural	3 (14)
Village	4 (18)
Town	10 (46)
City	4 (18)
Not disclosed	1 (5)

### Caregiver-reported HRQoL, sleep and relationships

**Fig. 1:**
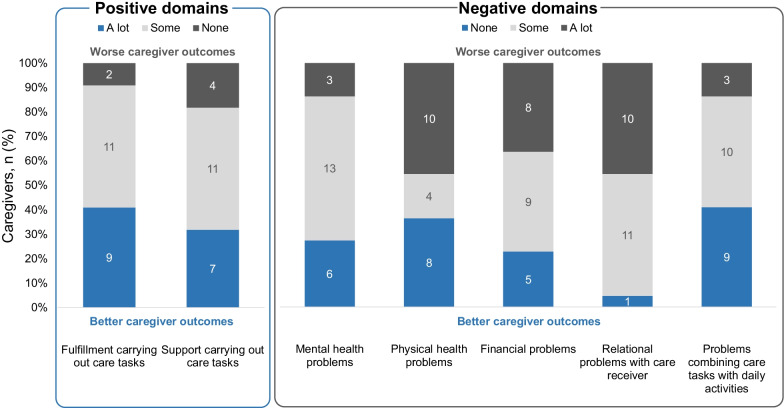
Caregiver-reported HRQoL by dimension of CarerQoL-7D (n = 22)

The mean CarerQoL-7D score was 63.7 (SD 24.5) out of 100, with a median score of 67.7. The mean CarerQoL-VAS score of general happiness was 5.7/10 (SD, 2.1). For the positive dimensions of the CarerQoL-7D, most caregivers appeared to indicate fulfilment in their caregiving responsibilities and having support available to help when needed (Fig. 1). In parallel, the majority of caregivers reported at least some problems across all negative domains related to their mental and physical health, finances, relationships, and ability to perform daily activities.

Responses to the CarerQoL-7D and CarerQoL-VAS, as well as other care-related quality of life items were also stratified by the patient’s PFIC type (no formal statistical comparison employed, due to sample size). Both CarerQoL-7D and CarerQoL-VAS scores were lower for patients with PFIC 2 when compared with PFIC 1, suggesting worse HRQoL outcomes for this subgroup (mean 59.4 vs. 71.2, and VAS score 5.3 vs. 6.5—Additional file 1: Table S3). More caregivers indicated impact on sleep in the PFIC 2 subgroup versus PFIC 1—93 versus 75%; caregiver mental health during the COVID-19 pandemic was also affected for a larger proportion in this subgroup (71%) compared to PFIC 1 caregivers (38%).

An additional stratum was defined by history of PFIC-related surgical procedures. When stratified according to surgical history (history of PBD/liver transplant surgeries vs. no PBD/liver transplant), and without formal statistical comparisons, CarerQoL-7D and CarerQoL-VAS scores both appeared higher among those whose children had none of these surgeries (mean 67.7 vs. 59.0/100 and 6.2 vs. 5.2/10, respectively).

**Fig. 2:**
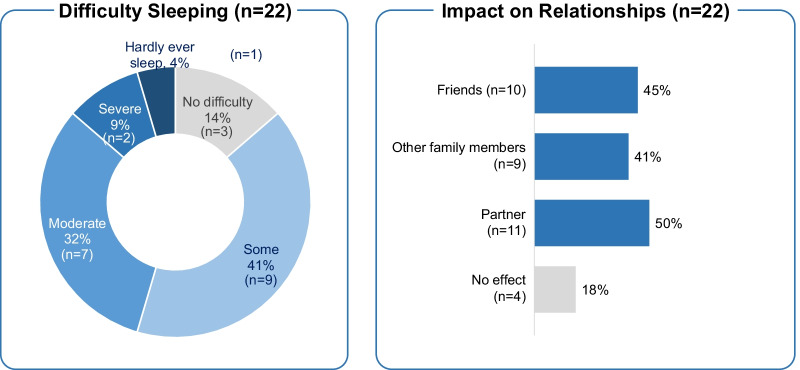
Impact of caregiving on sleep and personal relationships—captured with questions specifically developed for the PICTURE study

Additionally, more caregivers of children with PBD/liver transplant surgeries appeared to report difficulty with partner relationships; other HRQoL measures were generally comparable between subgroups (Additional file 1: Table S4). Nearly all caregivers reported an impact of caregiving responsibilities on sleeping (86%) and on relationships with partners, family, and/or friends (82%; Fig. 2). The impact on sleep and relationships was measured via questions specifically developed for this cohort of patients.

Beyond immediate help with caregiving tasks, no caregivers reported having any formal care support (such as professional assistance). Based on responses collected between September 2020 and March 2021, approximately half of caregivers reported an impact of the COVID-19 pandemic on resource use (10, 46%), and on their own mental health (13, 59%) and physical health (9, 41%).

### Caregiver productivity and activity impairment

The majority of caregivers indicated current employment (73%, n = 16). During the 7 days prior to data collection, mean impact on productivity due to absenteeism was reported as 27% (Table 2). Mean impact due to presenteeism was 40% (n = 13), resulting in an overall work productivity impairment (based on absenteeism and presenteeism) of 46% (n = 13). All caregivers responded to the regular activity impairment dimension of the WPAI, indicating a mean impairment of 49% in regular activities, due to caregiving over the past 7 days.

**Table 2:** Impact of caregiving on WPAI and professional career

Caregiver outcomes (n = 22)	N (%)	Mean (SD)
WPAI		
Currently working for pay, n (%)	16 (73)	–
Absenteeism, mean (SD)	16 (73)	26.9 (40.1)
Presenteeism, mean (SD)	13 (59)	40.0 (24.2)
Overall work productivity impairment, mean (SD)	13 (59)	45.6 (25.6)
Daily activity impairment, mean (SD)	22 (100)	48.6 (27.7)
Employment status, n (%)		–
Full-time employed	8 (36)	
Part-time employed	4 (18)	
Self-employed	4 (18)	
Homemaker	2 (9)	
Other	4 (18)	
Impact on work, n (%)		
Yes, I have missed workdays	8 (36)	–
Workdays missed, mean (SD)	8 (36)	12.9 (19.30)
No days missed, but productivity issues	5 (23)	–
No impact	3 (14)	–
Not applicable	6 (27)	–
Impact on stopping work, n (%)		
Yes, I have missed work years	8 (36)	–
Work years missed, mean (SD)	8 (36)	2.8 (1.5)
No years missed	12 (55)	–
Not applicable	2 (9)	–
Impact on career, n (%)		–
Impact of PFIC on career building	11 (50)	
Alternate career choice	5/11 (46)	
Prevented career progression	8/11 (73)	
Prevented from working more hours	8/11 (73)	
Other	6/11 (55)	

*PFIC* progressive familial intrahepatic cholestasis, *SD* standard deviation

A substantial proportion of caregivers (36%) reported suffering an impact on their employment, with a mean loss of 12.9 days (SD 19.3) within the last 3 months. Additionally, 36% of caregivers reported missing years of employment as a result of their caring duties, with a mean of 2.8 years (SD 9.5) during their career (Table 2). Half of caregivers (50%; n = 11) reported an impact of caregiving on career building, including limitations on work hours (73%), career progression (73%), and career choice (46%).

Through stratifying WPAI responses and productivity-related answers by PFIC type, differences become apparent in terms of impact of absenteeism (mean impact of 43% for PFIC 2 vs. 0% in PFIC 1), as well as daily activity impairment (mean impact of 54% in PFIC 2 vs. 40% in PFIC 1). On the other hand, scores for presenteeism and overall work productivity impairment were comparable across subgroups.

When compared to PFIC 1, a larger proportion of PFIC 2 caregivers reported missing workdays, and a substantially higher number of workdays were missed by PFIC 2 caregivers in the 3 months prior to data collection (16 days vs. 3 days in PFIC 1 group). A higher proportion in this subgroup also reported missing work years due to caregiving duties (43% vs. 25% for PFIC 2 and PFIC 1, respectively). The mean number of years missed was similar across subgroups (3.0 and 2.7 for PFIC 1 and PFIC2, respectively).

History of surgical procedures was also found to yield differences across strata in the assessment of productivity and activity impairment. Caregivers of patients with PBD/liver transplant surgeries reported greater impact on productivity due to absenteeism when compared to caregivers with no history of these surgeries (mean impact of 48.3% vs. 5.5%). Additionally, more caregivers in the PBD/liver transplant subgroup reported missing workdays (60% vs. 17%), and they appeared to miss substantially more workdays in the 3 months prior to data collection (mean, 15.8 vs. 4.0). Caregivers in this subgroup often reported missing years of work due to caregiving duties (70% vs. 8%; Additional file 1: Table S4). However, better scores from the daily activity impairment component of WPAI were observed among this subgroup (44%), when compared to caregivers of children who had not undergone PBD or liver transplantation (53%; Additional file 1: Table S4).

## Discussion

This analysis of humanistic burden from the multinational PICTURE study suggests PFIC has a meaningful impact on caregiver reported HRQoL, work productivity and performance of daily activities. While nearly all caregivers in the PICTURE study reported some level of fulfilment from taking care of their child with PFIC (91%), nearly all also reported caregiving-related mental (73%) and physical (64%) health problems, and relational problems with their child (95%). Despite having general support for caregiving tasks when needed (82%), caregivers reported disruption of close personal relationships (82%), particularly with their partner (50%). All caregivers reported impediments to accomplishing regular daily activities; on average, a level of impairment of 49% was reported, with nearly all caregivers (86%) suffering from some level of sleep difficulty attributable to the burden of caregiving. The majority of respondents were mothers aged 30–49 years—prime working age adults—nearly all of whom had a minimum of high school-level education (82%). While most caregivers were employed in some capacity (73%), only one-third were employed full-time (36%; 55% including self-employed) with half of the working caregivers reporting having missed an average of 13 workdays in the past 3 months, and losing, on average, 2.8 work years of their career. Absenteeism and presenteeism were high, with the majority of employed caregivers reporting limitations to career choice and progression (both 73%).

When considering outcomes from the perspective of presence/absence of surgeries in patient sample, it appears HRQoL scores of parents with surgically treated children are worse (CarerQoL mean scores of 59 in ‘surgeries’ group vs. 67 in ‘no surgeries’—no formal statistical comparison employed due to sample size). An observation by the clinical expert group overseeing the PICTURE study suggests this may be correlated with children in the ‘surgeries’ group having a more severe or more progressive form of PFIC 1 or 2, warranting a higher and potentially earlier need for life-saving surgeries. Furthermore, by looking at the PFIC type stratum, caregivers of PFIC 1 children have better HRQoL than PFIC 2 (CarerQoL mean scores of 71.2 in PFIC 1 group vs. 59 in PFIC 2)—which could possibly be correlated with higher rate and need of liver transplant in PFIC 2 cohorts, as stated by the currently available literature. Taken together, these findings provide a first overview of the burden of caregiving for a child with PFIC, with no other published reports of this burden to the authors’ best knowledge.

Given there is no available literature to provide direct context for these findings, the authors suggest cystic fibrosis (CF) may serve as a fair analogous condition for PFIC, as it also presents in early life and children/adolescents with CF also risk serious morbidity with demanding care and treatment regimens that substantially impact their HRQoL [23].

Fitzgerald and colleagues [16] reported caregiver burden using the CarerQoL-7D (with applied UK utility weights) among 195 families of children with CF, with a similar proportion of respondents having a university degree or higher (68–54% among mothers and fathers; PICTURE study, 64% overall) and being employed (79–90%; PICTURE, 73%). Parents of children with CF also reported comparable levels of fulfilment in performing their caregiving tasks for their children (115/130, 88%), as in our study (91%). Notably, CarerQoL-7D utility scores were lower (worse) for caregivers in the PICTURE study (median, 67.7) than among mothers and fathers in the CF study (median 84.7 and 89.2, respectively), though indirect comparisons should be interpreted with caution due to differences in sample sizes and disease nature. Approximately half of CF caregivers reported some level of financial problems due to care tasks (54%; 70/130), whilst a larger proportion of PFIC caregivers reported such financial impacts as an outcome of caregiving duties (17/22; 77%).

Significant predictors of high caregiver burden in the CF study included older age of the child with CF, being the mother of the child with CF, and having a child with a positive test (ever) for *Pseudomonas aeruginosa* (a prominent driver of morbidity and mortality) [16, 24]. Though analysis of predictive effects was not performed in our study due to its sample size, similar predicting factor investigations could be of interest in the context of PFIC, to enhance understanding of the drivers of caregiver burden.

This study also raises research questions which the authors recommend for investigation in future studies; among these, further insights into the potential correlation of the patients’ HRQoL with the caregivers’ HRQoL would likely aid the patient and medical community in identifying any remaining unmet needs.

Our findings should be interpreted in the context of the rare nature of PFIC and the consequent small sample size, which constitutes an unpreventable study limitation. While we were able to sample treating physicians and received recruiting support through PFIC-specific organizations, a clinical algorithm applied post-hoc was necessary to increase the investigators’ confidence that included cases were PFIC, rather than another manifestation of cholestasis. Nonetheless, the voluntary nature of participation and consequent potential for selection bias, both among the healthcare providers and caregivers, should be noted. Caregivers completing the questionnaires may have been subject to recall bias, particularly for measures intended to characterize outcomes over longer time periods in the past, though there were few such questions.

## Conclusions

The PICTURE study data indicated that caring for an individual with PFIC could cause comprehensive and meaningful burden on caregivers. Despite fulfilment from caregiving, the breadth and depth of these responsibilities reduced caregiver reported HRQoL including mental and physical health, productivity, career prospects, sleep, relationships and finances. The nature of PFIC and currently scarce available management approaches are potentially the reasons for the heavy burden on caregivers participating in this study, which may be alleviated by novel transformative therapeutic options. Given the level of unmet need that this study has uncovered, along with the scarcity of developed and published literature in this rare disease field, it is evident that more research is required to support PFIC patients and their caregivers.

## Supplementary Information

**Additional file 1**. An overview of the algorithm applied to the PICTURE patient dataset, the tariffs used to derive caregiver quality of life, and the characteristics and outcomes of the sample analyzed in this paper.

## Data Availability

The datasets analyzed during the current study are held under license by the University of Chester and are not publicly available, but will be available from the corresponding author on reasonable request, after 12 months from the publication date.

## Abbreviations

PFIC: Progressive familial intrahepatic cholestasis
ESLD: End-stage liver disease
ERG: Expert reference group
HRQoL: Health-related quality of life
Peds-QL: Pediatric quality of life inventory
PICTURE: Progressive familial intrahepatic cholestasis disease burden of illness: quantifying the socio-economic burden in the US, UK, France and Germany
eCRF: Electronic case report form
PFIC network: PFIC advocacy and resource network
CLDF: Children’s liver disease foundation
CarerQoL-7D: Care related quality of life-7 dimensions
CarerQoL-VAS: Care related quality of life-visual analogue scale
WPAI: Work productivity and activity impairment questionnaire
PBD: Partial biliary diversion
SD: Standard deviation
CF: Cystic fibrosis
