# Supplementary material for: Impact of progressive familial intrahepatic cholestasis on caregivers: caregiver-reported outcomes from the multinational PICTURE study
Source: Orphanet J Rare Dis. 2022 Feb 2;17:32. doi: 10.1186/s13023-022-02177-0 (PMC8809495; doi:10.1186/s13023-022-02177-0)
Supplement: Supplementary file 1 — Additional file 1. An overview of the algorithm applied to the PICTURE patient dataset, the tariffs used to derive caregiver quality of life, and the characteristics and outcomes of the sample analyzed in this paper. [file 13023_2022_2177_MOESM1_ESM.docx]

Impact of progressive familial intrahepatic cholestasis on caregivers: Caregiver-reported outcomes from the multinational PICTURE study

ADDITIONAL FILE 1

Figure S1. Clinical algorithm rules to identify PFIC patients


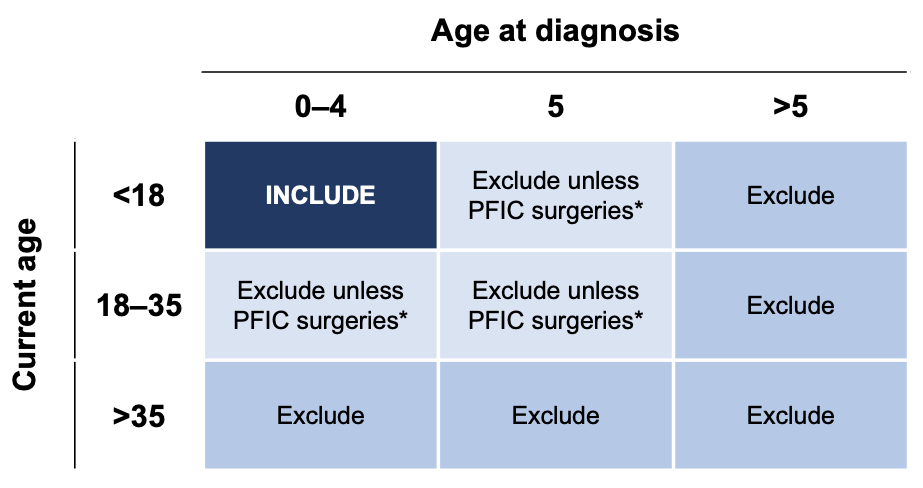
*Records of PFIC procedures such as biliary diversion or liver transplantation.

Table S1. CarerQol-7D tariffs for UK

| Dimension | Tariff for score | | |
| --- | --- | --- | --- |
|  | No | Some | A lot |
| Fulfilment | 0.0 | 10.1 | 13.0 |
| Relational problems | 12.0 | 8.8 | 0.0 |
| Mental health problems | 23.9 | 16.5 | 0.0 |
| Problems combining daily activities | 7.4 | 3.1 | 0.0 |
| Financial problems | 17.2 | 11.5 | 0.0 |
| Support | 0.0 | 6.9 | 8.1 |
| Physical health problems | 18.4 | 15.0 | 0.0 |

Table S2. Characteristics of patients with PFIC from the caregiver HRQoL sample

| **Characteristic, n (%)** | **Patients (n=22)** |
| --- | --- |
| Age  Mean, years  0-4  5-9  10-17  18-24 | 8.2  9 (41)  4 (18)  8 (36)  1 (5) |
| Sex  Female  Male | 13 (59)  9 (41) |
| Country  Germany  United Kingdom  United States | 8 (36)  7 (32)  7 (32) |
| PFIC type  PFIC 1  PFIC 2 | 8 (36)  14 (64) |

PFIC, progressive familial intrahepatic cholestasis.

Table S3. Caregiver outcomes stratified by patient’s PFIC type

| **Mean (SD) score unless noted** | **PFIC 1**  **(n=8)** | **PFIC 2**  **(n=14)** | **TOTAL**  **(n=22)** |
| --- | --- | --- | --- |
| CarerQoL-7D | 71.2 (18.3) | 59.4 (27.1) | 63.7 (24.5) |
| CarerQoL-VAS | 6.5 (1.8) | 5.3 (2.2) | 5.7 (2.1) |
| WPAI  Working for pay, n (%)  Absenteeism  Presenteeism  Overall work productivity impairment  Daily activity impairment | 6 (75)  0.0 (0.0)  45.0 (20.7)  45.0 (20.7)  40.0 (24.5) | 10 (71)  43.1 (43.7)  35.7 (27.6)  46.0 (30.8)  53.6 (29.0) | 16 (73%)  26.9 (40.1)  40.0 (24.2)  45.6 (25.6)  48.6 (27.7) |
| Impact on caregiver sleep, n (%)  No difficulty  Some difficulty  Moderate difficulty  Severe difficulty  Hardly ever sleep | 2 (25)  3 (37)  2 (25)  1 (12)  0 (0) | 1 (7)  6 (42)  5 (35)  1 (7)  1 (7) | 3 (14%)  9 (41%)  7 (32%)  2 (9%)  1 (4%) |
| Impact on caregiver relationships, n (%)  Relationship with partner  Relationships with other family members  Relationships with friends | 5 (63)  3 (38)  4 (50)  4 (50) | 13 (93)  8 (57)  5 (36)  6 (43) | 18 (82)  11 (50)  9 (41)  10 (45) |
| Employment status, n (%)  Full-time  Part-time  Self-employed  Homemaker  Other | 3 (38)  1 (13)  2 (25)  0 (0)  2 (25) | 5 (36)  3 (21)  2 (14)  2 (14)  2 (14) | 8 (36.4%)  4 (18.2%)  4 (18.2%)  2 (9.1%)  4 (18.2%) |
| Impact on work, n (%)  Yes I have missed workdays  Workdays missed, mean (SD)  No days missed, had productivity issues  No impact  Not applicable | 2 (25)  3.0 (2.8)  2 (25)  2 (25)  2 (25) | 6 (43)  16.2 (21.6)  3 (21)  1 (7)  4 (29) | 8 (36.4%)  12.9 (19.3)  5 (22.7%)  3 (13.6%)  6 (27.3%) |
| Impact on stopping work, n (%)  Yes I have missed work years  Work years missed, mean (SD)  No years missed  Not applicable | 2 (25)  3.0 (2.8)  5 (63)  1 (12) | 6 (43)  2.7 (1.2)  7 (50)  1 (7) | 8 (36.4%)  2.8 (1.5)  12 (54.5%)  2 (9.1%) |
| Impact on career, n (%)  Impact of PFIC on career building  Yes, alternate career choice  Yes, prevented career progression  Yes, prevented from working more hours  Yes, other | 3 (38)  1/3 (33)  2/3 (67)  2/3 (67)  1/3 (33) | 8 (57)  4/8 (50)  6/8 (75)  6/8 (75)  5/8 (63) | 11 (50)  5/11 (45)  8/11 (73)  8/11 (73)  6/11 (54) |
| COVID-19 impact  Resource use  Caregiver mental health  Caregiver physical health | 4 (50)  3 (38)  4 (50) | 6 (43)  10 (71)  5 (36) | 10 (45.5%)  13 (59.1%)  9 (40.9%) |

Table S4. Caregiver outcomes stratified by patient’s overall PFIC surgical history

| **Mean (SD) score unless noted** | **Overall without PBD/liver transplant surgeries (n=12)** | **Overall with PBD/liver transplant surgeries (n=10)** | **TOTAL**  **(n=22)** |
| --- | --- | --- | --- |
| CarerQoL-7D | 67.7 (22.3) | 59.0 (27.3) | 63.7 (24.5) |
| CarerQoL-VAS | 6.2 (1.6) | 5.2 (2.6) | 5.7 (2.1) |
| WPAI  Working for pay, n (%)  Absenteeism  Presenteeism  Overall work productivity impairment  Daily activity impairment | 8 (67)  5.5 (9.8)  42.5 (22.5)  44.9 (24.2)  52.5 (23.4) | 8 (80)  48.3 (48.0)  36.0 (28.8)  46.7 (30.6)  44.0 (32.7) | 16 (73%)  26.9 (40.1)  40.0 (24.2)  45.6 (25.6)  48.6 (27.7) |
| Impact on caregiver sleep, n (%)  No difficulty  Some difficulty  Moderate difficulty  Severe difficulty  Hardly ever sleep | 2 (17)  5 (42)  3 (25)  2 (17)  0 | 1 (10)  4 (40)  4 (40)  0  1 (10) | 3 (14%)  9 (41%)  7 (32%)  2 (9%)  1 (4%) |
| Impact on caregiver relationships, n (%)  Relationship with partner  Relationships with other family members  Relationships with friends | 10 (83)  4 (33)  5 (42)  6 (50) | 8 (80)  7 (70)  4 (40)  4 (40) | 18 (82)  11 (50)  9 (41)  10 (45) |
| Employment status, n (%)  Full-time  Part-time  Self-employed  Homemaker  Other | 4 (33)  2 (17)  2 (17)  1 (8)  3 (25) | 4 (40)  2 (20)  2 (20)  1 (10)  1 (10) | 8 (36.4%)  4 (18.2%)  4 (18.2%)  2 (9.1%)  4 (18.2%) |
| Impact on work, n (%)  Yes I have missed workdays  Workdays missed, mean (SD)  No days missed, had productivity issues  No impact  Not applicable | 2 (17)  4.0 (4.2)  4 (33)  2 (17)  4 (33) | 6 (60)  15.8 (21.8)  1 (10)  1 (10) 2 (20) | 8 (36.4%)  12.9 (19.3)  5 (22.7%)  3 (13.6%)  6 (27.3%) |
| Impact on stopping work, n (%)  Yes I have missed work years  Work years missed, mean (SD)  No years missed  Not applicable | 1 (8)  2.0 (NE)  9 (75)  2 (17) | 7 (70)  2.9 (1.6)  3 (30)  0 | 8 (36.4%)  2.8 (1.5)  12 (54.5%)  2 (9.1%) |
| Impact on career, n (%)  Impact of PFIC on career building  Yes, alternate career choice  Yes, prevented career progression  Yes, prevented working more hours  Yes, other | 2 (17)  0/2 (0)  1/2 (50)  0/2 (0)  1/2 (50) | 9 (90)  5/9 (56)  7/9 (78)  8/9 (89)  5/9 (56) | 11 (50)  5/11 (45)  8/11 (73)  8/11 (73)  6/11 (54) |
| COVID-19 impact  Resource use  Caregiver mental health  Caregiver physical health | 4 (33)  5 (42)  5 (42) | 6 (60)  8 (80)  4 (40) | 10 (45.5%)  13 (59.1%)  9 (40.9%) |

LT, liver transplantation; NE, not estimable; PBD, partial biliary diversion; PFIC, progressive familial intrahepatic cholestasis; SD, standard deviation.

Table S5. Mean (SD) CarerQoL-7D responses stratified by specific PBD/liver transplant surgical history

| **Mean (SD) score** | **Overall**  **(n=22)** | **PFIC surgical history** | | | |
| --- | --- | --- | --- | --- | --- |
|  |  | No LT/PBD  (n=12) | With LT  (n=6) | With PBD  (n=2) | With LT and PBD  (n=2) |
| CarerQoL-7D | 63.7 (24.5) | 67.7 (22.3) | 55.5 (21.7) | 58.9 (52.0) | 69.7 (36.8) |
| CarerQoL-VAS | 5.7 (2.1) | 6.2 (1.6) | 4.7 (2.3) | 5.0 (4.2) | 7.0 (2.8) |

LT, liver transplantation; PBD, partial biliary diversion; PFIC, progressive familial intrahepatic cholestasis; SD, standard deviation.

Table S6. Work productivity and activity impairment stratified by patient PFIC surgical history

|  | **Overall**  **(n=22)** | **PFIC surgical history** | | | |
| --- | --- | --- | --- | --- | --- |
|  |  | No LT/PBD  (n=12) | With LT  (n=6) | With PBD  (n=2) | With LT and PBD  (n=2) |
| Currently working for pay, n (%) | 16 (73) | 8 (67) | 4 (67) | 2 (100) | 2 (100) |
| Absenteeism, mean (SD) | 26.9 (40.1) | 5.5 (9.8) | 66.7 (47.1) | 50.0 (70.7) | 10.0 (14.1) |
| Presenteeism, mean (SD) | 40.0 (24.2) | 42.5 (22.5) | 55.0 (7.1) | 10.0 (NE) | 30.0 (42.4) |
| Overall work productivity impairment, mean (SD) | 45.6 (25.6) | 44.9 (24.2) | 71.7 (16.5) | 10.0 (NE) | 40.0 (28.3) |
| Daily activity impairment, mean (SD) | 48.6 (27.7) | 52.5 (23.4) | 48.3 (35.4) | 40.0 (42.4) | 35.0 (35.4) |

LT, liver transplantation; NE, not estimable; PBD, partial biliary diversion; PFIC, progressive familial intrahepatic cholestasis; SD, standard deviation.
